# Supplementary material for: Enhancing action recognition in educational settings using AI-driven information systems for public health monitoring
Source: Front Public Health. 2025 Jul 14;13:1592228. doi: 10.3389/fpubh.2025.1592228 (PMC12301300; doi:10.3389/fpubh.2025.1592228)
Supplement: Supplementary file 1 [file Data_Sheet_1.pdf]

Supplementary Material

0.1 Preliminaries

To establish a rigorous foundation for the integration of Artificial Intelligence (AI) in education, we begin by defining key mathematical formulations that model learning dynamics, student interactions, and knowledge progression. AI-driven educational frameworks leverage these formulations to adaptively personalize learning experiences, optimize instructional strategies, and improve student outcomes.

Let $\mathcal{S}$ represent the set of students, $\mathcal{C}$ denote the set of educational content items, and $\mathcal{T}$ be the set of time steps in a learning session. Each student interacts with educational content dynamically over time, and their learning process can be captured using mathematical models.

A student's knowledge state at time t is represented as a vector $\mathbf{k}_s^t \in \mathbb{R}^d$, where d denotes the dimensionality of the knowledge representation. The knowledge state evolves as students engage with learning materials and respond to exercises. This progression is modeled by the following transition function:

$$\mathbf{k}_s^{t+1} = f(\mathbf{k}_s^t, c_t, r_t), \tag{S1}$$

where $c_t \in \mathcal{C}$ is the educational content provided at time t, and $r_t \in [0, 1]$ represents the student's response to the learning activity, typically indicating correctness.

To assess a student's ability to answer questions correctly based on their knowledge state, we employ a probabilistic model using the logistic function. The probability of a correct response is given by:

$$P(r_t = 1|\mathbf{k}_s^t, c_t) = \sigma(\mathbf{k}_s^t \cdot \mathbf{v}_{c_t}), \tag{S2}$$

where $\mathbf{v}_{c_t} \in \mathbb{R}^d$ is the vector representation of the educational content, and $\sigma(x) = \frac{1}{1+e^{-x}}$ is the sigmoid activation function. This function ensures that the probability remains within the range of $[0, 1]$, reflecting the likelihood of a correct response based on a student's knowledge alignment with the content.

To optimize the instructional process, we define an objective function aimed at maximizing the expected knowledge gain of students over a learning session. The goal is to enhance knowledge retention and conceptual understanding through personalized content delivery. Formally, this is represented as:

$$\max_{\pi} \sum_{t=1}^{T} \mathbb{E}[G_t|\pi], \tag{S3}$$

where G_t represents the knowledge gain function at time step t, measuring the improvement in student understanding, and π denotes the adaptive instructional policy that selects optimal learning materials.

AI-driven educational systems utilize recommendation models to personalize learning materials by dynamically selecting content that best matches a student's current knowledge state. Given a student's knowledge representation $\mathbf{k}_s^t$, the optimal content recommendation is determined by:

$$c_t^* = \arg\max_{c\in\mathcal{C}} \mathbb{E}[P(r_t = 1|\mathbf{k}_s^t, c)]. \tag{S4}$$

To further refine the optimization process, we introduce a reinforcement learning formulation where the reward function is aligned with student engagement and long-term knowledge retention. Specifically, the optimal policy π^* is determined by maximizing the cumulative reward over an instructional period:

$$\pi^* = \arg\max_{\pi} \mathbb{E}\left[\sum_{t=1}^{T} R_t|\pi\right], \tag{S5}$$

where R_t denotes the reward signal at time t, encapsulating student performance, engagement, and knowledge acquisition.

0.2 Adaptive Knowledge Embedding Network (AKEN)

In this section, we introduce the Adaptive Knowledge Embedding Network (AKEN), a novel AI-driven model designed to enhance personalized learning in educational systems. The model captures dynamic student learning behaviors, predicts future performance, and optimizes content recommendations. Unlike conventional approaches, AKEN integrates a hierarchical embedding structure with recurrent updates to effectively model student knowledge evolution.

Dynamic Knowledge State Update

Let $\mathcal{S}$ be the set of students, $\mathcal{C}$ be the set of content items, and $\mathcal{T}$ be the learning timeline. Each student $s \in \mathcal{S}$ is associated with a knowledge state $\mathbf{k}_s^t \in \mathbb{R}^d$ at time t, which evolves over time as they engage with educational materials. The content items are embedded into a latent space, where each content item $c \in \mathcal{C}$ is represented as $\mathbf{v}_c \in \mathbb{R}^d$. The student's learning process is dictated by an interaction function that updates the knowledge state dynamically:

$$\mathbf{k}_s^{t+1} = \mathbf{k}_s^t + \Delta\mathbf{k}_s^t, \tag{S6}$$

where $\Delta\mathbf{k}_s^t$ denotes the knowledge increment at time t, determined by content engagement and student interaction. The change $\Delta\mathbf{k}_s^t$ is modeled using a gated recurrent update mechanism:

$$\Delta\mathbf{k}_s^t = \mathbf{z}_s^t \odot \tilde{\mathbf{k}}_s^t, \tag{S7}$$

where $\mathbf{z}_s^t$ serves as an update gate that controls the extent of information retention, and $\tilde{\mathbf{k}}_s^t$ represents the candidate knowledge state that incorporates new learning experiences. The update gate $\mathbf{z}_s^t$ is computed using a sigmoid activation function:

$$\mathbf{z}_s^t = \sigma(W_z[\mathbf{k}_s^t, \mathbf{v}_{c_t}, r_t] + b_z), \tag{S8}$$

where W_z represents a coefficient matrix, b_z represents an offset parameter, and r_t represents the student's response to content c_t. The candidate knowledge state $\tilde{\mathbf{k}}_s^t$ is computed using a hyperbolic tangent activation:

$$\tilde{\mathbf{k}}_s^t = \tanh(W_k[\mathbf{k}_s^t, \mathbf{v}_{c_t}, r_t] + b_k). \tag{S9}$$

To capture personalized learning dynamics, the response r_t may depend on previous performance, engagement history, and cognitive factors. A reinforcement-based update mechanism can be introduced, where the weight of knowledge acquisition is adjusted dynamically. The overall student knowledge state is influenced by cumulative interactions over time, and the final update is constrained to ensure stability and prevent knowledge degradation.

Performance Prediction Mechanism

The probability of a correct response is estimated as:

$$P(r_t = 1|\mathbf{k}_s^t, c_t) = \sigma(\mathbf{k}_s^t \cdot \mathbf{v}_{c_t}). \tag{S10}$$

This prediction function provides a real-time estimation of student progress, facilitating adaptive learning by guiding instructional decisions. The vector $\mathbf{k}_s^t$ represents the student's knowledge state at time t, while $\mathbf{v}_{c_t}$ denotes the feature representation of content item c_t. The function $\sigma(\cdot)$ is the sigmoid activation, ensuring the probability remains between 0 and 1.

To enhance learning, instructional content should be selected to optimize the student's progress. The optimal content selection strategy aims to maximize the expected learning outcome:

$$c_t^* = \arg\max_{c \in \mathcal{C}} \mathbb{E}[P(r_t = 1|\mathbf{k}_s^t, c) + \alpha G_t]. \tag{S11}$$

Here, G_t represents the expected knowledge gain when content c is presented, and α is a balancing parameter that weighs the immediate likelihood of a correct response against the long-term knowledge improvement.

To model the knowledge state evolution, an update function is introduced based on student performance:

$$\mathbf{k}_s^{t+1} = \mathbf{k}_s^t + \eta(r_t - P(r_t = 1|\mathbf{k}_s^t, c_t))\mathbf{v}_{c_t}. \tag{S12}$$

This equation adjusts the knowledge state using a learning rate η, refining it according to the discrepancy between actual response r_t and predicted probability.

The knowledge gain G_t can be estimated using an expected improvement function:

$$G_t = \sum_{c \in \mathcal{C}} P(r_t = 1|\mathbf{k}_s^t, c) \cdot \Delta\mathbf{k}_s^t(c), \tag{S13}$$

where $\Delta\mathbf{k}_s^t(c)$ represents the expected change in knowledge state upon practicing content c. This formulation integrates the probabilistic model with knowledge dynamics to support intelligent content sequencing.

An additional refinement incorporates content difficulty d_c and individual adaptability, modifying the probability estimation as:

$$P(r_t = 1|\mathbf{k}_s^t, c_t) = \sigma(\mathbf{k}_s^t \cdot \mathbf{v}_{c_t} - d_{c_t}). \tag{S14}$$

This accounts for inherent difficulty levels, ensuring that the system can personalize recommendations based on both student ability and content complexity.

Engagement-Aware Learning Adaptation

To effectively capture student engagement in learning systems, we introduce an engagement state e_s^t, which evolves over time based on prior engagement levels, knowledge states, and contextual factors. This dynamic engagement evolution is modeled as:

$$P(e_s^{t+1}|e_s^t, \mathbf{k}_s^t, c_t) = h(e_s^t, \mathbf{k}_s^t, c_t), \tag{S15}$$

where $h(\cdot)$ is a trainable function designed to capture engagement fluctuations influenced by cognitive and external factors. The function adapts based on student interactions, enabling personalized learning interventions.

The knowledge state of a student, denoted as $\mathbf{k}_s^t$, plays a crucial role in learning performance and engagement. We assume a probabilistic update mechanism for knowledge evolution:

$$P(\mathbf{k}_s^{t+1}|\mathbf{k}_s^t, e_s^t, q_t) = g(\mathbf{k}_s^t, e_s^t, q_t), \tag{S16}$$

where $g(\cdot)$ captures how knowledge transitions occur due to engagement levels and question difficulty q_t. Higher engagement leads to better knowledge retention, reinforcing the importance of engagement-aware learning strategies.

To predict student performance, we define the response probability $\hat{r}_t$ as:

$$\hat{r}_t = \sigma(f(\mathbf{k}_s^t, e_s^t, q_t)), \tag{S17}$$

where $\sigma(\cdot)$ is the sigmoid function ensuring outputs in $[0, 1]$, and $f(\cdot)$ models the dependency of response accuracy on knowledge state, engagement, and task difficulty. This formulation allows adaptation of learning paths based on both knowledge mastery and motivation.

To optimize the Adaptive Knowledge and Engagement Network (AKEN), we employ a composite loss function:

$$\mathcal{L} = -\sum_{s \in \mathcal{S}} \sum_{t=1}^{T} [r_t \log \hat{r}_t + (1 - r_t) \log(1 - \hat{r}_t)] + \lambda \|\theta\|^2. \tag{S18}$$

This loss function balances engagement-aware performance modeling with regularization to prevent overfitting. The parameter λ controls the strength of regularization, ensuring stable model learning.

Furthermore, engagement influences learning rates dynamically. We define the adaptive learning rate as:

$$\eta_t = \eta_0(1 + \alpha e_s^t), \tag{S19}$$

where η_0 is the base learning rate, and α scales the effect of engagement. A higher engagement state results in a larger effective learning rate, allowing quicker adaptation to new knowledge.

0.3 Dynamic Personalized Learning Strategy (DPLS)

To further enhance the effectiveness of AI-driven education, we propose the Dynamic Personalized Learning Strategy (DPLS), an adaptive learning mechanism designed to optimize instructional sequences and maximize student engagement. Unlike traditional static learning paths, DPLS dynamically adjusts content delivery based on real-time student performance and engagement levels, ensuring an individualized learning experience.

Adaptive Content Recommendation

To personalize learning and optimize knowledge acquisition, we define an adaptive content recommendation system that selects the next learning content c_t^* for a student $s \in \mathcal{S}$ based on their knowledge state $\mathbf{k}_s^t$. The objective is to maximize long-term learning gains:

$$c_t^* = \arg\max_{c \in \mathcal{C}} \mathbb{E}[R_t + \gamma V(\mathbf{k}_s^{t+1})|\mathbf{k}_s^t, c], \tag{S20}$$

where R_t represents the immediate reward reflecting student correctness, $\gamma \in (0, 1]$ is a discount factor governing future rewards, and $V(\mathbf{k}_s^{t+1})$ is a value function estimating the expected future learning benefits.

The recommendation system operates within a Markov Decision Process (MDP) framework, where a student's knowledge state $\mathbf{k}_s^t$ evolves over time as they engage with different learning contents. Each state transition follows:

$$P(\mathbf{k}_s^{t+1}|\mathbf{k}_s^t, c_t, r_t) = g(\mathbf{k}_s^t, c_t, r_t), \tag{S21}$$

where $g(\cdot)$ is a trainable function modeling knowledge evolution based on student interactions and correctness feedback.

The policy $\pi(c|\mathbf{k}_s)$, governing content recommendations, is optimized through a reward function incorporating both accuracy and engagement:

$$J(\pi) = \mathbb{E} \sum_{t=1}^{T} \left[R_t + \beta E_t\right], \tag{S22}$$

where E_t represents an engagement-related reward, and β controls the trade-off between academic success and motivation.

To enhance adaptivity, we introduce a dynamic learning rate α_t that adjusts based on a student's recent performance:

$$\alpha_t = \alpha_0 \cdot \exp\left(-\lambda \sum_{\tau=t-w}^{t} R_\tau\right), \tag{S23}$$

where α_0 is an initial learning rate, λ is a decay factor, and w determines the window size for performance assessment. A lower recent performance results in a slower learning rate to provide additional reinforcement.

Content sequencing is guided by a curriculum difficulty function $D(c, \mathbf{k}_s^t)$, ensuring a balance between challenge and comprehension:

$$D(c, \mathbf{k}_s^t) = \mathbb{E}[1 - P(R_t = 1|\mathbf{k}_s^t, c)], \tag{S24}$$

where a higher difficulty corresponds to a lower expected correctness probability. The system adaptively selects content to maintain an optimal difficulty level, preventing frustration or stagnation.

Explainable Learning Decisions

To enhance transparency in AI-driven education, we integrate an explainability module that provides intuitive feedback to students and instructors. The contribution of each knowledge component to a prediction is measured using:

$$\phi_j = \frac{\partial P(r_t = 1|\mathbf{k}_s^t, c_t)}{\partial k_{s,j}^t}, \tag{S25}$$

where ϕ_j quantifies the importance of the j-th knowledge feature in determining the likelihood of a correct response r_t. This sensitivity analysis allows educators to interpret model decisions, diagnose potential learning gaps, and tailor interventions accordingly.

To further enhance the explainability of the model, we introduce a cumulative importance score:

$$\Phi_s^t = \sum_{j=1}^{d} w_j \phi_j, \tag{S26}$$

where w_j represents a weight factor adjusting for the significance of different knowledge components. This aggregated metric provides an overall measure of how knowledge components contribute to student performance, enabling a more holistic understanding of learning progress.

Since engagement is crucial for effective learning, we incorporate an engagement predictor:

$$e_s^{t+1} = \rho(e_s^t, \mathbf{k}_s^t, c_t), \tag{S27}$$

where $\rho(\cdot)$ models engagement fluctuations based on prior engagement e_s^t, knowledge state $\mathbf{k}_s^t$, and content characteristics c_t. Engagement trends guide content adaptation to sustain student motivation.

To refine engagement predictions, we introduce an engagement gradient metric:

$$\Delta e_s^t = \frac{\partial e_s^{t+1}}{\partial c_t}. \tag{S28}$$

This gradient measures the sensitivity of engagement to content modifications, aiding in dynamically adjusting instructional material. Higher values of Δe_s^t indicate that engagement is significantly affected by content variations, prompting real-time curriculum adaptation.

We define an adaptive content difficulty adjustment mechanism:

$$c_{t+1} = \lambda(c_t, \Phi_s^t, e_s^t), \tag{S29}$$

where $\lambda(\cdot)$ updates content difficulty based on student knowledge importance scores and engagement levels. This ensures that students receive appropriately challenging content to optimize their learning trajectory.

Efficient Adaptive Assessment

To minimize redundant assessments while ensuring accurate student knowledge estimation, we employ an adaptive assessment mechanism that dynamically selects the most informative assessment item at each step. The selection process is formulated as:

$$a_t^* = \arg\max_{a \in \mathcal{A}} I(\mathbf{k}_s^t; r_a), \tag{S30}$$

where $I(\mathbf{k}_s^t; r_a)$ quantifies the information gain derived from administering assessment a at time step t for student s. This information-theoretic approach allows for the reduction of testing fatigue by prioritizing assessments that contribute the most to knowledge estimation.

To further refine the estimation of student proficiency, we incorporate a Bayesian update model:

$$P(\mathbf{k}_s^{t+1}|r_t, \mathbf{k}_s^t) \propto P(r_t|\mathbf{k}_s^t)P(\mathbf{k}_s^t), \tag{S31}$$

where the posterior knowledge state $\mathbf{k}_s^{t+1}$ is computed using the prior state $\mathbf{k}_s^t$ and the response r_t to the administered assessment. This Bayesian formulation ensures that each student's knowledge estimate evolves dynamically as more data is gathered.

The overall learning objective for the Dynamic Personalized Learning System (DPLS) is given by:

$$\mathcal{L} = \sum_{s \in \mathcal{S}} \sum_{t=1}^{T} \left[-r_t \log \hat{r}_t + \lambda \|\theta\|^2 - \beta E_t \right], \tag{S32}$$

where the first term represents the negative log-likelihood of student responses, ensuring that the model accurately predicts responses. The second term $\lambda\|\theta\|^2$ serves as a regularization term to prevent overfitting, while the third term βE_t integrates engagement-aware learning, where E_t denotes an engagement metric at time t.

To ensure a balance between knowledge acquisition and engagement, we introduce a reinforcement learning formulation, where the reward function is designed as:

$$R_t = \alpha I(\mathbf{k}_s^t; r_t) + \gamma E_t - \delta C_t. \tag{S33}$$

Here, α controls the weight of information gain, γ scales the engagement term, and δ penalizes cognitive load C_t to prevent overwhelming the student.

Moreover, to maintain consistency in difficulty adaptation, we introduce an adaptive difficulty scheduling mechanism:

$$d_t = d_{t-1} + \eta \cdot \text{sgn}(\hat{r}_t - r_t), \tag{S34}$$

where d_t represents the difficulty level of the next assessment, updated based on the discrepancy between predicted and actual responses. The learning rate η controls the adjustment speed, ensuring a smooth progression in assessment difficulty.
